# Annotations

**Published:** 1897-07-03

**Authors:** 


### ANNOTATIONS.

### The Plague on the Red Sea.

Several cases of plague are reported to have occurred at Jeddah on the Red Sea, and very elaborate precautions are being taken to prevent its spread. It must be remembered, however, that, although during the last few years much excitement has been caused by the outbreaks of plague which have occurred in China, and especially in India, the disease is almost constantly present far nearer home. In Persia and Kurdestan, on the shores of the Caspian, on the Volga, in Mesopotamia, and even in Arabia itself, plague has over and over again broken out with great virulence during the past twenty years. So far as infection is concerned, there have been opportunities in plenty for the development of epidemics in Arabia and Asia Minor. But plague is a very peculiar complaint. The history of the outbreak in India has but shown again what was already well known, that the social and sanitary conditions of a population are far more important factors in the production of an epidemic than any amount of infection. The disease has picked out certain spots, and has attained to virulence in certain places; but it has missed other spots equally open to it, and it has in a most marked degree passed over certain groups of people living in the midst of the epidemic area and notoriously exposed to the infection. It would seem that for the production of an outbreak the coincidence of several factors is necessary. Certain climatic conditions, an accumulation of filth, dirty habits, the introduction of infection, and, apparently, the presence of a sufficient number of susceptible people to enable the contagious material to develop rapidly the full intensity of its virulence, would seem to be the assemblage of conditions requisite for the development of an epidemic. Inspection of travellers, then, is useful as a means of preventing the spread of infection, and it may even be admitted that a rigid quarantine, such as only Orientals will submit to, may in exceptional circumstances prevent its entry, at any rate for a short time; but the real point at which plague is vulnerable is in the fact that it cannot develop except in the presence of filthy surroundings. It is by eliminating dirt and securing good sanitation that we can best break up the combination without which plague cannot successfully attack; while in the presence of filth and foul living, other factors, over which we have no control, may at any time make the occurrence of plague not only a possibility but a grave and very real danger.

### By Whom are Hair-dyes Used?

Who are the people who chiefly buy hair-dyes? Most people will answer, "Fading beauties, who see in their first grey hairs the threatened termination of their empire; women of fashion who insist on their gowns being of the tint that is in the latest vogue, and must therefore colour their hair and complexion to harmonise with it if they are to present a pleasing appearance; actresses whose counterfeit presentment of the part they play must be complete in every detail." So far as the widely-advertised "restorers" of the fashion journals go this surmise would be correct; but the chemists in the poorer quarters of London could tell another tale. There the chief purchasers of hair-dye are not women but men, and it is not vanity that prompts them to hide the signs of age. In the incessant competition that goes on among the unskilled, the younger claimants, who are presumably the stronger, are preferred, and grey hairs may mean starvation. It is pitiful that such a dread of old age should hang over many of our fellows. Pitiful, too, that at an age when with our politicians, our scientists, our lawyers and doctors, we reckon them in their prime, the artisan should be condemned as "past work." But, as the body ages sooner than the mind, it is inevitable that this should be so wherever man contributes more of brute strength than of intelligence to the performance of his task. The clever man has become an overseer by the age his fellow is dismissed, and looks forward to years of usefulness. Therefore, let us pray that men may more and more come to contribute the intelligent, and not the mechanical, force to our industries. Machinery of steel that is moved by steam wears out painlessly, and can be renewed without cruelty; but the machinery of flesh and blood!—God help it when not even hair-dye can conceal its deficiencies!

### Rosebery House.

In an old quarter of Edinburgh, picturesque but unfashionable, and till lately dirty, a colony of social reformers have taken up their abode. They have built cleanly and sanitary houses, in some of which they live themselves, in families, or in coteries of earnest men and women, who try to enter into brotherly relations with those of the poor and labouring classes who live round them. In the midst of this colony a new house is at this moment being erected, a tall mansion, with turrets in the old Scottish baronial style, and decorated over one window with an earl's coronet and the letter R., carved in stone. This is Lord Rosebery's new house, the Edinburgh home of the Ex-Premier, the Lord of Dalmeny, of the Durdans, of Mentmore. Ragged children are playing round it as it rises; the owner, when he occupies it, will certainly have an unsurpassed view of the Firth of Forth, but he will also be within hearing of the bugle-call of the garrison at Edinburgh Castle, and more closely within hearing of riots on Saturday nights. He will have his abode in the people's quarter, and will have the chance, as few of his order have, of learning how the people look at life. The theory of those who first formed the colony is that the conditions of modern life, which separate class from class, are false and wrong, that our slums and our aristocratic quarters are alike an abomination. Brotherly relations should not be impossible between rich and poor, and would not be if they came into contact, and realised each other's labours, anxieties, and responsibilities. For the revolutionist it is a good thing to keep the classes apart, for then each will hate the other; one will envy, one will despise; and hatred, contempt, and envy lead to murder now, as in the days of Cain. But for the reformer it is a good thing to bring classes together, that they may understand each other, may help and advise each other, and work together for the good of all. If this be possible it can be only by both classes meeting each other, not now and then on formal occasions, but naturally, day after day. For the statesman who desires the good of the people the knowledge thus gained is beyond all price, and we may reasonably hope that what the master of Rosebery House learns in the Lawn Market of Edinburgh may be used to the profit of the whole British nation.

